# Diagnosis of nasal and eye allergies: the Allergies, Immunotherapy, and RhinoconjunctivitiS (AIRS) patient survey

**DOI:** 10.1186/1710-1492-10-S1-A59

**Published:** 2014-03-03

**Authors:** Michael Blaiss, Mark Dykewicz, Bryan Leatherman, David Skoner, Nancy Smith, Felicia Allen-Ramey

**Affiliations:** 1 Allergy & Asthma Care, Germantown, TN, 38138, USA; 2 Wake Forrest Baptist Health, Winston-Salem, NC, 27157, USA; 3 Coastal ENT Associates, Gulfport, MISS, 39501, USA; 4 West Penn Allegheny Health System, Cranberry, PA, 16066, USA; 5 Merck & Co, Inc, West Point, PA 19486, USA

## Background

Knowledge and avoidance of allergies can lead to better allergy symptom control. The AIRS study assessed approaches to diagnosis of nasal and eye allergies or allergic rhinoconjunctivitis in the US.

## Methods

A national sample of 34,030 households using a dual frame approach of random digit dialing and cell phone sample were targeted. Patients aged =5 years with a health care professional diagnosis of hay fever, allergic rhinitis, rhino-conjunctivitis, nasal or eye allergies and symptoms or medication for condition in past 12 months were surveyed. Data on specific diagnosis and allergy testing were collected.

## Results

Based on screening land-line sample of 20,835 households, 18% of individuals were diagnosed with one of conditions of interest. Of the 2765 surveyed patients, 86% were diagnosed with nasal allergies, 59%, hay fever, 54% eye allergies, 30%, allergic rhinitis and 13%, rhinoconjunctivitis. Four percent of respondents reported they were given an allergy test by doctor or health professional in past four months, 3% in past year, 7%, 1-2 years ago, and 37%, 3 or more years ago. Of those, 71% reported that they had a skin prick test, 13% had blood test and 12% had both blood and skin prick test. 47% report they never received an allergy test.

## Conclusions

The AIRS survey demonstrated that 18% of individuals > 5 years were diagnosed with an above allergic condition. Although these respondents had been diagnosed by a health care professional with “allergies”, almost half never had any allergy testing to determine the triggers of their condition.

